# Optimized respiratory‐resolved motion‐compensated 3D Cartesian coronary MR angiography

**DOI:** 10.1002/mrm.27208

**Published:** 2018-04-22

**Authors:** Teresa Correia, Giulia Ginami, Gastão Cruz, Radhouene Neji, Imran Rashid, René M. Botnar, Claudia Prieto

**Affiliations:** ^1^ School of Biomedical Engineering and Imaging Sciences King's College London London United Kingdom; ^2^ MR Research Collaborations, Siemens Healthcare Limited Frimley United Kingdom; ^3^ Escuela de Ingeniería, Pontificia Universidad Católica de Chile Santiago Chile

**Keywords:** compressed sensing, coronary MRA, image navigator, respiratory motion compensation

## Abstract

**Purpose:**

To develop a robust and efficient reconstruction framework that provides high‐quality motion‐compensated respiratory‐resolved images from free‐breathing 3D whole‐heart Cartesian coronary magnetic resonance angiography (CMRA) acquisitions.

**Methods:**

Recently, XD‐GRASP (eXtra‐Dimensional Golden‐angle RAdial Sparse Parallel MRI) was proposed to achieve 100% scan efficiency and provide respiratory‐resolved 3D radial CMRA images by exploiting sparsity in the respiratory dimension. Here, a reconstruction framework for Cartesian CMRA imaging is proposed, which provides respiratory‐resolved motion‐compensated images by incorporating 2D beat‐to‐beat translational motion information to increase sparsity in the respiratory dimension. The motion information is extracted from interleaved image navigators and is also used to compensate for 2D translational motion within each respiratory phase. The proposed Optimized Respiratory‐resolved Cartesian Coronary MR Angiography (XD‐ORCCA) method was tested on 10 healthy subjects and 2 patients with cardiovascular disease, and compared against XD‐GRASP.

**Results:**

The proposed XD‐ORCCA provides high‐quality respiratory‐resolved images, allowing clear visualization of the right and left coronary arteries, even for irregular breathing patterns. Compared with XD‐GRASP, the proposed method improves the visibility and sharpness of both coronaries. Significant differences (*p* < .05) in visible vessel length and proximal vessel sharpness were found between the 2 methods. The XD‐GRASP method provides good‐quality images in the absence of intraphase motion. However, motion blurring is observed in XD‐GRASP images for respiratory phases with larger motion amplitudes and subjects with irregular breathing patterns.

**Conclusion:**

A robust respiratory‐resolved motion‐compensated framework for Cartesian CMRA has been proposed and tested in healthy subjects and patients. The proposed XD‐ORCCA provides high‐quality images for all respiratory phases, independently of the regularity of the breathing pattern.

## 1 INTRODUCTION

Coronary MRA (CMRA) is a promising noninvasive imaging technique for the detection of coronary artery disease. However, one of the major challenges in free‐breathing whole‐heart 3D CMRA is image degradation due to respiratory motion. Diaphragmatic navigator‐gated and tracked acquisitions are commonly used to minimize blurring and ghosting artifacts caused by respiratory motion.^1^ Nevertheless, this approach only compensates for translational motion in the superior–inferior (SI) direction and suffers from low scan efficiency, leading to prolonged acquisition times, because only data within a small (end‐expiration) respiratory gating window are accepted. Moreover, motion within this window is estimated from the right hemi‐diaphragmatic displacement during breathing, which has a more pronounced movement than the heart; hence, a scaling factor of 0.6 is commonly applied.^2^ However, this value is not suitable for all subjects and cardiac regions and may also vary between inspiration and expiration.^3^

Recently, several approaches have been proposed to achieve about 100% scan efficiency (no data rejection) and compensate for respiratory‐induced cardiac motion. These approaches usually correct for beat‐to‐beat translational motion based on one‐dimensional self‐navigation^4^, ^5^, ^6^ or 2D and 3D image navigators.^7^, ^8^, ^9^, ^10^, ^11^, ^12^ In the former, the SI translational displacement of the heart due to respiration is estimated directly from the CMRA data. Hence, motion correction is still limited to one dimension, and contribution from static tissues (e.g., chest wall) may affect the motion estimation performance. The latter methods make use of low‐spatial resolution images, immediately preceding the imaging sequence, to extract and correct for 2D or 3D beat‐to‐beat translational respiratory motion. To account for (more complex) 3D motion, respiratory binning approaches have also been proposed to “freeze” respiratory motion. These approaches sort the CMRA data or image navigators (iNAVs) into multiple respiratory phases (or “bins”) and generate respiratory‐resolved or (nonrigid) motion‐corrected images.^13^, ^14^, ^15^, ^16^, ^17^, ^18^, ^19^, ^20^, ^21^

Respiratory‐resolved approaches enable the study of pathophysiological interactions between the cardiovascular and respiratory systems.^22^ This is particularly important for certain types of congenital heart and pericardial disease, in which respiratory‐related variations in systemic venous return impact cardiac morphology and function.^23^, ^24^ Recently, XD‐GRASP (eXtra‐Dimensional Golden‐angle RAdial Sparse Parallel MRI) has been proposed to achieve 100% scan efficiency and provide respiratory‐resolved 3D radial CMRA images.^25^ This approach reconstructs the respiratory phase images by exploiting total variation (TV) sparsity along the respiratory dimension. However, for Cartesian liver imaging, XD‐GRASP has been shown to suffer from reduced image quality,^26^ especially for phases with large respiratory displacements. Cartesian sampling presents several advantages over radial sampling, such as higher SNR efficiency, lower sensitivity to off‐resonance and gradient delays, and reduced reconstruction complexity. However, in the presence of motion, Cartesian imaging suffers from ghosting artifacts, whereas radial imaging is more robust to motion since the central region of k‐space is densely sampled and the k‐space center is sampled multiple times.^27^, ^28^

Here, a robust framework for Cartesian imaging is proposed to provide a high‐quality motion‐compensated image of the coronary arteries, alongside respiratory temporal information (i.e., good‐quality respiratory‐resolved motion‐compensated CMRA images), which can provide useful additional physiological information. The proposed Optimized Respiratory‐resolved Cartesian Coronary MR Angiography (XD‐ORCCA) reconstruction incorporates motion information from iNAVs to increase the sparsity in the respiratory dimension. This approach is similar to motion‐adaptive temporal domain techniques that have been proposed to increase sparsity in the cardiac motion direction for breath‐hold 2D dynamic cine MRI, in which motion is estimated from intermediate cardiac motion‐resolved images.^29^, ^30^, ^31^ Furthermore, 2D beat‐to‐beat translational motion information extracted from iNAVs is used to minimize intrabin motion in XD‐ORCCA. The proposed XD‐ORCCA was tested on 10 healthy subjects and 2 patients with cardiovascular disease, and compared against XD‐GRASP.

## 2 METHODS

Whole‐heart 3D CMRA data are acquired using a prototype Cartesian trajectory, which samples the k_y_‐k_z_ plane using a golden‐step Cartesian trajectory with spiral profile ordering.^32^ A low‐resolution 2D iNAV (spatially encoded into the ramp‐up pulses of the balanced SSFP [bSSFP] sequence) is acquired in every heartbeat, before each spiral interleaf of the whole‐heart 3D CMRA acquisition.^7^ The 2D iNAVs are registered to a common respiratory position (end‐expiration) to estimate 2D beat‐to‐beat translational (left–right [LR] and SI) motion. The 2D translational motion parameters are obtained through intensity‐based image registration, using mutual information as a similarity measure and steepest gradient descent optimization. Then, the estimated SI motion is used to group the 3D CMRA data into 5 equally populated respiratory bins. The 2D translational motion correction within each bin is performed in k‐space before the reconstruction to improve the image quality of each bin. This step involves correcting the 3D CMRA data within each bin to the corresponding average bin position. This also provides an estimate of interbin translational motion, which is used during the reconstruction to align intrabin motion‐corrected images to a reference respiratory position, as a way to increase sparsity in the respiratory domain.

### 2.1 Respiratory‐resolved XD‐ORRCA reconstruction

The XD‐GRASP method reconstructs each undersampled bin by solving the following optimization problem:^25^

$$\hat{x}=\;\text{arg}\;\underset{x}{\text{min}\;}\;\left\{\frac{1}{2}{\left\|\|Ex-\;k\|\right\|}_{2}^{2}+\;\alpha \;\Psi_{t}\left(x\right)\right\},$$

where $x=\;{\left[x_{1}{}^{T},\;x_{2}{}^{T},\cdots ,x_{N}{}^{T}\right]}^{T}$ is the respiratory‐resolved image series; $k=\;{\left[k_{1}{}^{T},\;k_{2}{}^{T},\cdots ,k_{N}{}^{T}\right]}^{T}$ are the binned k‐space data for N bins; $\Psi_{t}$ is the one‐dimensional temporal TV function; and α is a regularization parameter. The encoding operator E=AFS  incorporates the sampling matrix A for each bin b, Fourier transform F, and coil sensitivities S.

The XD‐ORCCA technique further improves sparsity in the respiratory dimension by incorporating 2D translational respiratory motion information in the temporal TV function. Moreover, intrabin 2D translational motion correction and TV spatial regularization are considered in XD‐ORCCA. Therefore, XD‐ORCCA respiratory‐resolved images are obtained by solving the following optimization problem:

$$\hat{x}=\text{arg}\;\underset{x}{\text{min}\;}\left\{\frac{1}{2}{\left\|\|Ex-b\|\right\|}_{2}^{2}+\;\alpha \;\Psi_{t}\left(Rx\right)+\beta \;\Psi_{s}\left(x\right)\right\},$$

where $b={\left[b_{1}{}^{T},\;b_{2}{}^{T},\cdots ,b_{N}{}^{T}\right]}^{T}$ are the 2D translational‐corrected binned k‐space data; $\Psi_{s}$ is the 3D spatial TV function;^33^ β is a regularization parameter; Rx is the motion‐corrected respiratory domain; and $\Psi_{t}\left(Rx\right)$ is the motion‐corrected temporal sparsity, which is given by

$$\Psi_{t}\left(Rx\right)=\;{\left\|\|\nabla_{t\;}\left(Rx\right)\|\right\|}_{1}={||[\begin{array}{ccccc} \;\;I\; & -I\; & \;0\; & \cdots & \;0\;\\ \;0\; & \;I\; & -I\; & \cdots \; & 0\\ \vdots & & \ddots & & \vdots \\ \;0\; & \;\cdots & \;0\; & \;I & -I\;\; \end{array}]\;\;\left[\begin{array}{c} T_{1}x_{1}\\ T_{2}x_{2}\\ \vdots \\ T_{N}x_{N}\; \end{array}]|\right|}_{1},$$

where $\nabla_{t\;}$ is the finite differences operator along the temporal respiratory dimension; **I** is the identity matrix; and $T_{b}$ is the translation transform that maps the bin image $x_{b}$ to the reference image $x_{1}$ (end‐expiration). Note that $T_{1}=I$. For a given vector u, the l1‐norm is defined as ${\left\|\|u\|\right\|}_{1}=\sum_{i=1}^{n}\left|u\left(i\right)\right|$, where *i* is the *i*th element of u and *n* is the total number of elements.

Both XD‐GRASP and XD‐ORCCA can be solved with the nonlinear conjugate gradient method.

### 2.2 In vivo experiments

In vivo free‐breathing experiments were performed on 10 healthy subjects and 2 patients on a 1.5T scanner (Magnetom Aera, Siemens Healthcare, Erlangen, Germany) with 18‐channel body and 32‐channel spine coils. The study was approved by the institutional review board (approval numbers 1/11/12 and 15/NS/0030), and written informed consent was obtained from all subjects before the scan. Two patients with cardiovascular disease agreed to 15 minutes of additional MR imaging after the clinical protocol. Patient 1 had impaired left ventricular systolic function due to a nonischemic cardiomyopathy. Patient 2 had known ischemic heart disease with previous stent deployment in the middistal segment of the right coronary artery (RCA) and suffered from frequent ectopic heartbeats.

Data were acquired with an electrocardiogram‐triggered 3D bSSFP sequence using the following parameters: coronal orientation, FOV = 320 × 320 × 80–104 mm^3^, resolution = 1 × 1 × 2 mm^3^ (1.2 × 1.2 × 1.2 mm^3^ for patients), TR/TE = 3.6/1.56 ms, flip angle = 90 °, T_2_ preparation = 40 ms, spectral presaturation with inversion recovery fat saturation, subject‐specific middiastolic trigger delay, acquisition window = 90–125 ms (corresponding to 25–35 k‐space readouts per heartbeat), 1 spiral interleaf per heartbeat, with acquisition time of 9–14 minutes. The 2D iNAVs were acquired using 14 coronal bSSFP startup echoes with the same FOV and orientation as the 3D CMRA acquisition, linearly increasing startup flip angles, 1 × 21.7 mm^2^ in‐plane resolution (interpolated to 1 × 1 mm^2^ during the reconstruction), and 80–104‐mm slice thickness. Patient acquisitions were performed approximately 25 minutes after gadolinium‐based contrast injection, which was required for clinical late gadolinium enhancement imaging.

For each 3D CMRA data set, respiratory‐resolved images were reconstructed using XD‐GRASP and the proposed XD‐ORCCA. Additionally, for 1 healthy subject, data were also reconstructed using XD‐GRASP with intrabin 2D translational correction (XD‐GRASP+TC), to illustrate the effect of incorporating motion correction into XD‐GRASP (see Supporting Information Figure S1 for a description of the methods). Furthermore, for 1 healthy subject, data were also reconstructed using XD‐ORCCA without TV spatial regularization to isolate the effect of the improved sparsity in the respiratory dimension. The regularization parameters were selected empirically and set at $\alpha =2\;\times \;10^{-5},\;\beta =7\;\times \;10^{-7}\;$ for all subjects.

Reconstructions were performed using MATLAB (MathWorks, Natick, MA) on a Linux PC with 32 Intel Xeon E5‐2680 central processing units at 2.70 GHz and 198 GB memory. For the image reconstruction step, the differences in computation time between XD‐GRASP and XD‐ORCCA were very small. The reconstruction time was about 5640 seconds for XD‐GRASP and about 5880 seconds for XD‐ORCCA. However, XD‐ORCCA required additional translational motion estimation, using 2D image registration, and correction of 3D CMRA binned k‐space data, taking about 105 seconds per respiratory phase. Hence, the total computation time of XD‐ORCCA was about 6405 seconds.

All reconstructed images were reformatted using “Soap‐bubble” to allow simultaneous visualization of the RCA and left anterior descending coronary artery (LAD).^34^ This software was also used to quantify the quality of the reconstructed images in terms of visible vessel length and sharpness (for the full length and first 4 cm) of both RCA and LAD. A paired t‐test was used to compare the quantitative results, and *p* < .05 was considered to indicate a significant difference.

## 3 RESULTS

**Figure 1:**
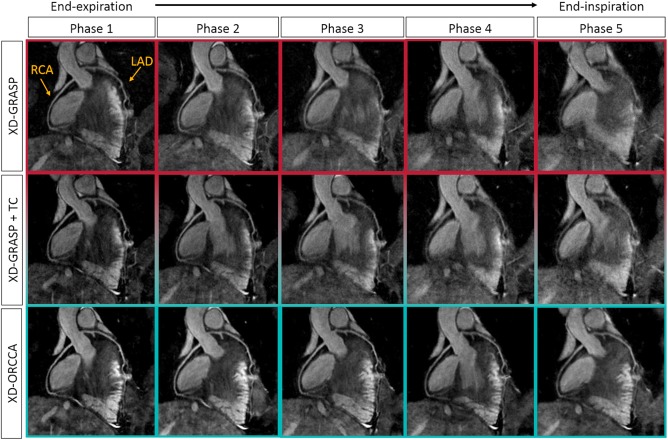
Reformatted respiratory‐resolved images obtained for 1 representative subject using XD‐GRASP (eXtra‐Dimensional Golden‐angle RAdial Sparse Parallel MRI; top), XD‐GRASP with intrabin motion correction (translational correction [TC]; middle), and the proposed XD‐ORCCA (Optimized Respiratory‐resolved Cartesian Coronary MR Angiography; bottom). For each method, 5 respiratory phases are shown: from end‐expiration (left) to end‐inspiration (right). Each image shows the right coronary artery (RCA) and the left anterior descending coronary artery (LAD). Including TC in XD‐GRASP slightly improved the quality of the respiratory‐resolved images. However, motion artifacts are still visible in phases 4 and 5. The XD‐ORCCA method considerably improves the vessel visibility and sharpness of both coronary arteries. Nevertheless, XD‐GRASP provides good‐quality end‐expiration images, which usually contain less respiratory motion. However, the coronary vasculature cannot be clearly visualized in near end‐inspiration phases

Whole‐heart reformatted respiratory‐resolved images for 1 representative healthy subject, displaying both the RCA and LAD, are shown in Figure 1. Images reconstructed using the XD‐GRASP, XD‐GRASP+TC, and XD‐ORCCA methods are included. The images show 5 respiratory phases, from end‐expiration (phase 1) to end‐inspiration (phase 5). The results show the visual quality improvements achieved by adding intrabin motion correction to XD‐GRASP and, in addition to it, combining motion correction with the proposed sparsifying transform in XD‐ORCCA. Respiratory binning is an efficient technique to reduce motion, particularly for data closest to end‐expiration, which usually contains less motion than data near end‐inspiration. Hence, respiratory phases near end‐inspiration usually exhibit more motion artifacts than near end‐expiration phases. The effect of remaining respiratory motion can be seen in Figure 1 for XD‐GRASP, where blurring is observed in phases 3 to 5. Incorporating iNAV‐based intrabin motion correction into XD‐GRASP slightly improves the quality of the respiratory‐resolved images, but motion artifacts are still visible in phases 4 and 5. The proposed XD‐ORCCA produces high‐quality respiratory‐resolved images for all respiratory phases, including those in which the displacement of the heart is the largest.

**Figure 2:**
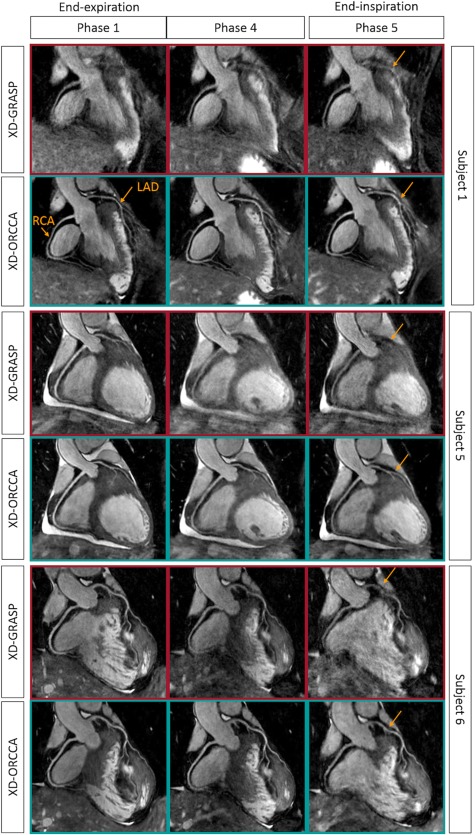
Reformatted respiratory‐resolved images obtained for 3 representative subjects using XD‐GRASP and XD‐ORCCA, showing the RCA and LAD. The images correspond to the respiratory phase that typically presents less respiratory motion (end‐expiration, phase 1) and the 2 respiratory phases with largest respiratory motion amplitude (phases 4 and 5, end‐inspiration). The XD‐GRASP method provides good‐quality end‐expiration images in the absence of residual intrabin motion (subjects 5 and 6), allowing visualization of both RCA and LAD. However, for more irregular breathers, XD‐GRASP fails to provide respiratory‐resolved images with sufficient quality to visualize the coronary tree (subject 1). The proposed XD‐ORCCA improves the visibility and sharpness of both coronary arteries for all respiratory phases, particularly for those that have more motion (arrows). Hence, Cartesian XD‐GRASP shows dependence on the regularity of the breathing pattern, whereas XD‐ORCCA provides a more consistent performance across subjects

Reformatted respiratory phase images (1, 4, and 5) obtained with XD‐GRASP and XD‐ORCCA from 3 healthy subjects are shown in Figure 2. The images correspond to the bin that typically presents less respiratory motion (end‐expiration, phase 1) and the 2 bins with largest respiratory motion (phases 4 and 5, end‐inspiration). The proposed XD‐ORCCA consistently produces high‐quality respiratory‐resolved images by increasing temporal sparsity (Supporting Information Figures S2 and S3). Motion blurring is observed in XD‐GRASP Cartesian images for subjects with more irregular breathing patterns, which is evident for subject 1. Nevertheless, for more regular breathing patterns, in which respiratory motion is relatively small, XD‐GRASP provides good‐quality end‐expiration images. However, the coronary tree cannot be clearly visualized in the phase 4 and phase 5 images. The breathing patterns of the subjects shown in Figure 2 can be seen in Supporting Information Figure S4.

**Figure 3:**
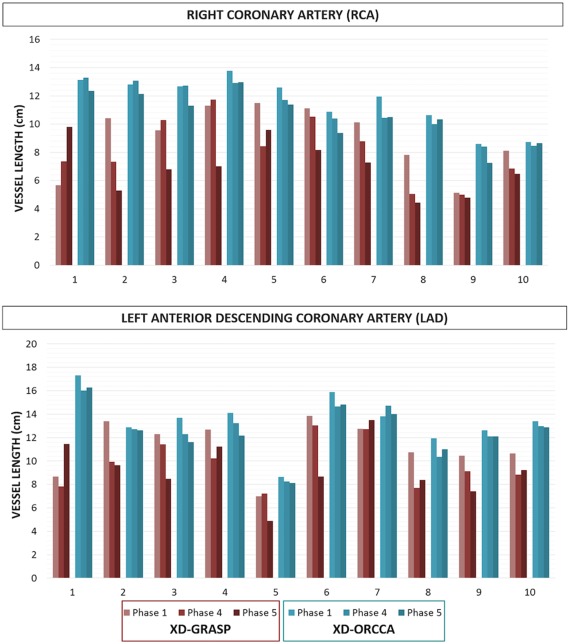
Vessel length of the RCA and LAD for all 10 healthy subjects, for phase 1 (end‐expiration), phase 4 and phase 5 (end‐inspiration), obtained using the XD‐GRASP and XD‐ORCCA approaches. Compared with XD‐GRASP, the proposed XD‐ORCCA improves the visible vessel length and reduces the variability across respiratory phases. Significant differences (*p* < .05) in visible vessel length were observed between XD‐GRASP and XD‐ORCCA for both coronary arteries (corresponding respiratory phases)

**Figure 4:**
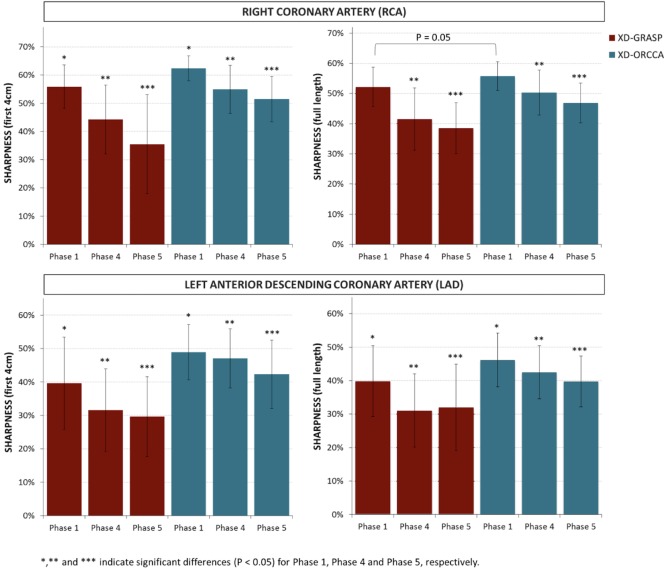
Vessel sharpness obtained from XD‐GRASP and XD‐ORCCA phase 1 (end‐expiration), phase 4 and phase 5 (end‐inspiration) images, for 10 healthy subjects. Image quality was assessed by measuring the vessel sharpness for the first 4 cm (left) and full length (right) of the RCA and LAD. Significant differences (*p* < .05) were found in vessel sharpness between XD‐GRASP and XD‐ORCCA for both coronaries. No significant differences were observed in the sharpness of the full length of the RCA in phase 1 images (indicated by *p* = .05). The proposed XD‐ORCCA provides images with improved vessel sharpness. Moreover, compared with XD‐GRASP, XD‐ORCCA reduces the variability across subjects and respiratory phases

The quality of phase 1, phase 4, and phase 5 images was quantitatively assessed in terms of visible vessel length and sharpness (first 4 cm and full length) of both RCA and LAD, for all 10 healthy subjects. The visible RCA and LAD vessel length measured from the images reconstructed with XD‐GRASP and XD‐ORCCA, for all 10 healthy subjects, is shown in Figure 3. The proposed XD‐ORCCA produces respiratory‐resolved images with improved visible RCA and LAD vessel length (Figure 3) and sharpness (Figure 4) compared with XD‐GRASP. The improvement in visible vessel length and sharpness achieved with XD‐ORCCA (Figures 2, 3, 4) is more evident for respiratory phases near end‐inspiration. Significant differences in visible vessel length were identified for both coronary arteries between the 2 methods. Additionally, a statistically significant difference was found in the sharpness of both coronary arteries between XD‐ORCCA and XD‐GRASP (Figure 4), except for the full length of the RCA in phase 1 images (*p* = .05).

**Figure 5:**
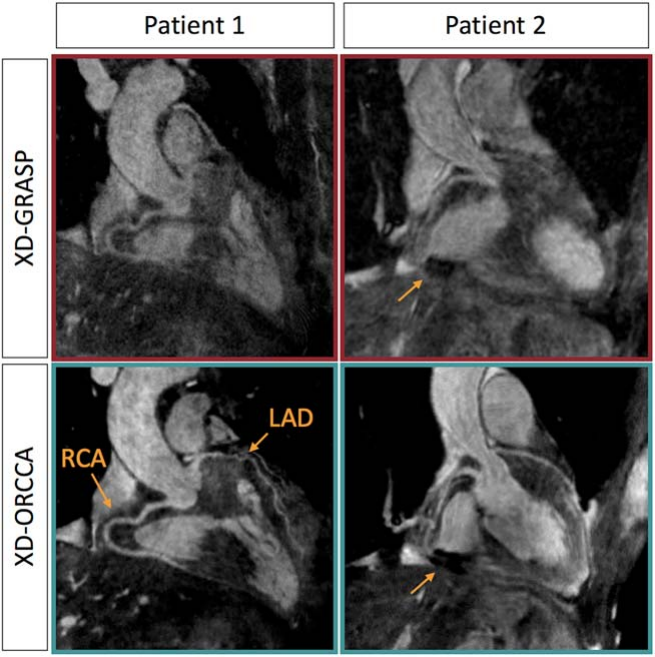
Reformatted images obtained using XD‐GRASP (top) and XD‐ORCCA (bottom) for 2 patients, showing the RCA and LAD. Only the respiratory phase with the highest quality is displayed. Patient 1 had impaired left ventricular systolic function due to a nonischemic cardiomyopathy. Patient 2 had a middistal RCA stent in situ and suffered from frequent ectopic heartbeats, and therefore cardiac motion presented an additional challenge. The proposed XD‐ORCCA recovers both coronary arteries and coronary stent (arrow) in patient 2. Irregular respiratory motion, respiratory drift, and large left–right motion amplitude severely deteriorate the delineation of the coronary arteries in XD‐GRASP Cartesian images. Conversely, XD‐ORCCA produces images that allow the visualization of very tortuous coronary arteries even for irregular breathing patterns

Figure 5 shows reformatted coronal views of XD‐GRASP and XD‐ORCCA images (respiratory phase with the highest quality), to visualize the RCA and LAD of 2 patients. Irregularities in the respiratory motion pattern of the patients (Supporting Information Figure S5) have a detrimental effect in XD‐GRASP Cartesian images. Conversely, XD‐ORCCA provides images that allow visualization of both coronary arteries, and coronary stent for patient 2, even for irregular breathing patterns.

For the healthy subjects, the maximum and average SI (LR) respiratory motion amplitudes were 22.27 mm (2.83 mm) and 12.99 ± 4.96 mm (2.14 ± 0.54 mm), respectively. For patient 1, the maximum SI (LR) value was 13.72 mm (3.85 mm). For patient 2, the corresponding value was 35 mm (6.5 mm).

## 4 DISCUSSION

A novel respiratory‐resolved motion‐compensated reconstruction method that achieves 100% scan efficiency has been proposed for free‐breathing 3D Cartesian CMRA. In the proposed XD‐ORCCA, CMRA data are distributed into multiple respiratory bins and 2D beat‐to‐beat translational motion is estimated from 2D iNAVs, which is used to correct for intrabin 2D translational motion in k‐space, thereby improving the image quality of each bin. During the reconstruction, intrabin motion‐corrected images are aligned to 1 respiratory position to increase sparsity in the respiratory dimension.

Recently, XD‐GRASP has been proposed to provide respiratory‐resolved images and achieve 100% scan efficiency, using primarily non‐Cartesian acquisitions and exploiting sparsity along the respiratory phase dimension. This method is suitable for Cartesian acquisitions, but it has been shown to suffer from reduced image quality,^26^ particularly in the presence of large intrabin respiratory motion amplitudes. Here, XD‐GRASP and XD‐ORCCA Cartesian respiratory‐resolved images (5 respiratory phases) were reconstructed for 10 healthy subjects and 2 patients with cardiovascular disease. The proposed XD‐ORCCA produces high‐quality respiratory‐resolved images for all respiratory phases, allowing clear visualization of both coronary arteries. The proposed XD‐ORCCA substantially reduces respiratory motion, thereby improving the visualized vessel length and sharpness of both coronaries compared with Cartesian XD‐GRASP. Significant differences in visible vessel length and sharpness were identified for both coronary arteries between XD‐ORCCA and XD‐GRASP. Differences were not significant only for the sharpness of the full length of the RCA in phase 1 images (end‐expiration).

Including intrabin translational motion correction into the reconstruction slightly improves the quality of the respiratory‐resolved images. The spatial TV regularization term only produces a denoising effect in the resulting reconstructions. Hence, the use of translational motion correction to increase the sparsity in the respiratory dimension appears to explain a significant part of the improvement in image quality. Motion blurring was observed in XD‐GRASP Cartesian images, particularly for respiratory phases in which the displacement of the heart is the largest (usually near end‐inspiration). However, for more regular breathing patterns, XD‐GRASP normally provides end‐expiration images with good quality, allowing visualization of both coronary arteries. Irregular breathing or respiratory drift, which is particularly common in patients,^35^ severely deteriorates the delineation of the coronary arteries in motion‐resolved XD‐GRASP Cartesian images, even for the highest‐quality respiratory phase images (bin with less motion). In addition, XD‐GRASP only considers SI motion, but LR motion can cause a substantial degradation in image quality. For example, the smallest respiratory bin widths obtained for patients 1 and 2 were 1 mm and 1.4 mm, respectively, but the corresponding LR motion amplitudes were 2.9 mm and 4.7 mm. Hence, Cartesian XD‐GRASP appears to be dependent on the regularity of the breathing pattern, whereas XD‐ORCCA provides a more consistent performance across subjects and respiratory phases.

The number of respiratory phases selected provides a good compromise between remaining intrabin motion and undersampling artifacts.^13^ However, better results could potenally be achieved by selecting the number of respiratory phases according to the (ir)regularity of the breathing pattern. For example, when the respiratory motion amplitude is relatively large, more respiratory phases could be added until all of the bin widths are smaller than a certain value (e.g., 3mm).^36^ Moreover, finite differences sparsity along the respiratory dimension (TV operator in the respiratory direction) could be calculated between every respiratory phase, $x_{b},$ and its 2 adjacent respiratory phases, $x_{b-1}$ and $x_{b+1}$, to exploit the temporal redundancies in both forward and backward directions (opposite to a unique direction with conventional TV regularization). This could be achieved by adding a temporal regularization term in the backward direction to the optimization problem.^29^ It would also require applying a translation transformation to map $x_{b-1}$ to $x_{b}$ and another transformation to map $x_{b+1}$ to $x_{b}$.^29^ In theory, this approach should provide a sparser representation of the motion‐corrected respiratory domain than designating the end‐expiration phase as the reference of the motion‐corrected domain, particularly because it depends less on the quality of the end‐expiration image and accuracy of the motion model.

An autofocus technique could also be applied to each bin to estimate iteratively the 3D translation parameters that maximize the sharpness of the coronaries, using the estimated SI and LR motion as prior information, and further improve bin images.^20^, ^35^, ^37^, ^38^ Alternatively, intrabin residual translational motion may be addressed by acquiring 3D iNAVs to estimate beat‐to‐beat 3D translational motion, and thus additionally allowing for motion correction in the anterior–posterior direction.^39^ Subsequently, these bin images can be used to estimate 3D translational or nonrigid interbin motion parameters at each XD‐ORCCA iteration. These techniques should further increase the sparsity in the respiratory dimension, and hence improve the quality of the respiratory‐resolved reconstructions. Otherwise, the high‐quality respiratory‐resolved XD‐ORCCA images can be used to estimate 3D bin‐to‐bin nonrigid motion, which can be used to provide a single‐phase 3D nonrigid motion‐compensated image.^17^, ^33^ However, all of these iterative methods come at the expense of higher computational complexity.

The proposed XD‐ORCCA showed promising results in 2 patients, which presented irregular respiration, respiratory drift, and large (SI and LR) respiratory motion amplitudes. However, in future studies, this technique will be evaluated in a large cohort of patients with coronary artery disease and at risk of developing the disease. Moreover, future studies will aim to accelerate the acquisition and achieve submillimeter isotropic resolution.

Even though this study was focused on Cartesian imaging, XD‐ORCCA is also suitable for non‐Cartesian imaging (e.g., radial, stack‐of‐stars/spirals). A comparison between non‐Cartesian and Cartesian XD‐ORCCA will be the subject of a future study. Moreover, the concept of motion‐corrected alignment to increase sparsity of XD‐ORCCA can be also extended to the cardiac motion dimension. Extension of XD‐ORCCA to enable cardiac and respiratory‐resolved 3D CMRA will be studied as future work.

## 5 CONCLUSIONS

A robust and highly efficient respiratory‐resolved motion‐compensated framework for 3D Cartesian CMRA has been described. The proposed XD‐ORCCA exploits sparsity in a motion‐corrected domain to generate high‐quality respiratory‐resolved images. More specifically, 2D beat‐to‐beat translational motion is estimated from 2D iNAVs and incorporated into the XD‐ORCCA reconstruction to increase sparsity in the respiratory dimension. Additionally, this motion information is used to compensate for residual intrabin motion. The proposed XD‐ORCCA provides high‐quality respiratory‐resolved images for all respiratory phases, even in the presence of irregular breathing patterns or respiratory drifts, which often occurs in patients.

## Supporting information

Additional Supporting Information may be found in the supporting information tab for this article.

**FIGURE S1** Schematic representation of XD‐GRASP (top), XD‐GRASP with intrabin 2D translational motion correction (XD‐GRASP+TC) (middle), and XD‐ORCCA (bottom). The dots represent the respiratory signal extracted from the image navigators (iNAVs), which corresponds to the estimated superior–inferior (SI) translational motion. For all methods, this information is used to separate the 3D coronary MRA (CMRA) data into 5 different respiratory phases (or bins), to reconstruct respiratory‐resolved images. In XD‐GRASP, respiratory‐resolved images are reconstructed by exploiting total variation sparsity in the respiratory dimension. In XD‐GRASP+TC, the 3D CMRA data within each bin are corrected for 2D translational motion to the center of each bin, to reduce residual intrabin motion. In XD‐ORCCA, 2D translational motion correction within each bin is performed in k‐space before the reconstruction (as in XD‐GRASP+TC). Furthermore, intrabin motion‐corrected images (x_*b*_) are aligned (using the 2D translational transform T_*b*_) to 1 respiratory position (end‐expiration) to further increase sparsity in the respiratory dimension.
**FIGURE S2** Example of temporal sparsity achieved with XD‐GRASP (left) and XD‐ORCCA (right). The proposed XD‐ORCCA increases the sparsity in the respiratory dimension by incorporating translational motion information into the sparsifying operator along the temporal dimension. Translational information is extracted from 2D interleaved iNAVs.
**FIGURE S3** Reformatted respiratory‐resolved images obtained for 1 representative subject using XD‐GRASP (top), XD‐ORCCA without spatial TV regularization (middle), and XD‐ORCCA with spatial TV regularization (bottom). For each method, respiratory phases 1 (end‐expiration), 4, and 5 (end‐inspiration) are shown. Each image shows the RCA and the LAD. Including spatial TV regularization in XD‐ORCCA slightly improved the quality of the respiratory‐resolved images, because of its denoising effect. Hence, the use of translational motion to further increase the sparsity in the respiratory dimension appears to explain a significant part of the improvement in image quality of the proposed XD‐ORCCA in comparison to XD‐GRASP.
**FIGURE S4** Respiratory displacement values at each heartbeat, obtained from the 2D image navigators along the SI (left) and left–right (LR) (right) directions, for 3 representative subjects. The end‐expiration SI position is used as reference. The horizontal lines (SI plots) and colors represent the different respiratory phases. Subject 5 had a regular breathing pattern with small LR displacements (maximum of 1.23 mm). For this subject, XD‐GRASP provided a good end‐expiration image, as the SI bin width was 1.05 mm and the maximum LR displacement was 0.56 mm. However, for phases 4 and 5 the SI bin width was approximately 3 mm, and hence, blurring motion is visible in the corresponding XD‐GRASP images. Subject 6 presented a respiratory drift for the first 200 heartbeats. Nevertheless, the end‐expiration images obtained with XD‐GRASP have good quality, as the SI bin width was 0.81 mm and the maximum LR displacement for that bin was 0.29 mm. However, for the end‐inspiration phase, the SI bin width was 5.69 mm and the LR displacement was 2.07 mm. This large residual intrabin motion deteriorates the quality of end‐inspiration XD‐GRASP images. Subject 1 showed a more irregular breathing pattern and a substantial respiratory drift, which resulted in a large end‐expiration bin of 5.12 mm width. In addition, LR motion was present in all of the respiratory phases (about 1.6 mm). For this case, XD‐GRASP images were substantially degraded by respiratory motion. The XD‐ORCCA approach provided high‐quality respiratory‐resolved images even for subject 1, which had a more irregular breathing pattern. For all 10 subjects, the overall SI respiratory bin widths for phases 1 to 5 were 1.55 ± 1.35 mm, 1.17 ± 0.54 mm, 1.61 ± 0.91 mm, 2.78 ± 1.04 mm, and 5.88 ± 3.40 mm, respectively. The overall LR displacements for phases 1 to 5 were 1.01 ± 0.55 mm, 1.29 ± 0.56 mm, 1.16 ± 0.45 mm, 1.43 ± 0.50 mm, and 1.80 ± 0.64 mm, respectively.
**FIGURE S5** Respiratory displacement values at each heartbeat, obtained from the 2D image navigators along the SI (left) and LR (right) directions, for 2 patients with cardiovascular disease. The end‐expiration SI position is used as reference. The horizontal lines (SI plots) and colors represent the different respiratory phases. Patient 1 presented a large respiratory drift. Patient 2 had an irregular breathing pattern, a slight respiratory drift, and large SI motion displacements. For patients 1 and 2, the respiratory phase with the smallest SI displacement (phase 2) only had a maximum displacement of 1.07 and 1.37 mm, respectively. However, the corresponding LR displacements were 2.92 and 4.72 mm. Cartesian XD‐GRASP does not provide good‐quality images in the presence of residual intrabin respiratory motion, particularly if this motion is in the LR direction. The XD‐ORCCA method produces images that allow the visualization of the coronary arteries in both patients.
