# Supplementary material for: Optimized respiratory‐resolved motion‐compensated 3D Cartesian coronary MR angiography
Source: Magn Reson Med. 2018 Apr 22;80(6):2618–29. doi: 10.1002/mrm.27208 (PMC6220806; doi:10.1002/mrm.27208)
Supplement: Supplementary file 1 — FIGURE S1 Schematic representation of XD‐GRASP (top), XD‐GRASP with intrabin 2D translational motion correction (XD‐GRASP+TC) (middle), and XD‐ORCCA (bottom). The dots represent the respiratory signal extracted from the image navigators (iNAVs), which corresponds to the estimated superior–inferior (SI) translational motion. For all methods, this information is used to separate the 3D coronary MRA (CMRA) data into 5 different respiratory phases (or bins), to reconstruct respiratory‐resolved images. In XD‐GRASP, respiratory‐resolved images are reconstructed by exploiting total variation sparsity in the respiratory dimension. In XD‐GRASP+TC, the 3D CMRA data within each bin are corrected for 2D translational motion to the center of each bin, to reduce residual intrabin motion. In XD‐ORCCA, 2D translational motion correction within each bin is performed in k‐space before the reconstruction (as in XD‐GRASP+TC). Furthermore, intrabin motion‐corrected images (x b) are aligned (using the 2D translational transform T b) to 1 respiratory position (end‐expiration) to further increase sparsity in the respiratory dimension. FIGURE S2 Example of temporal sparsity achieved with XD‐GRASP (left) and XD‐ORCCA (right). The proposed XD‐ORCCA increases the sparsity in the respiratory dimension by incorporating translational motion information into the sparsifying operator along the temporal dimension. Translational information is extracted from 2D interleaved iNAVs. FIGURE S3 Reformatted respiratory‐resolved images obtained for 1 representative subject using XD‐GRASP (top), XD‐ORCCA without spatial TV regularization (middle), and XD‐ORCCA with spatial TV regularization (bottom). For each method, respiratory phases 1 (end‐expiration), 4, and 5 (end‐inspiration) are shown. Each image shows the RCA and the LAD. Including spatial TV regularization in XD‐ORCCA slightly improved the quality of the respiratory‐resolved images, because of its denoising effect. Hence, the use of translational mo [file MRM-80-2618-s001.pdf]

SUPPORTING INFORMATION

Optimized Respiratory-resolved Motion-compensated 3D Cartesian Coronary MR Angiography

T Correia,* G Ginami, G Cruz, R Neji, I Rashid, R M Botnar, C Prieto
*E-mail: teresa.correia@kcl.ac.uk

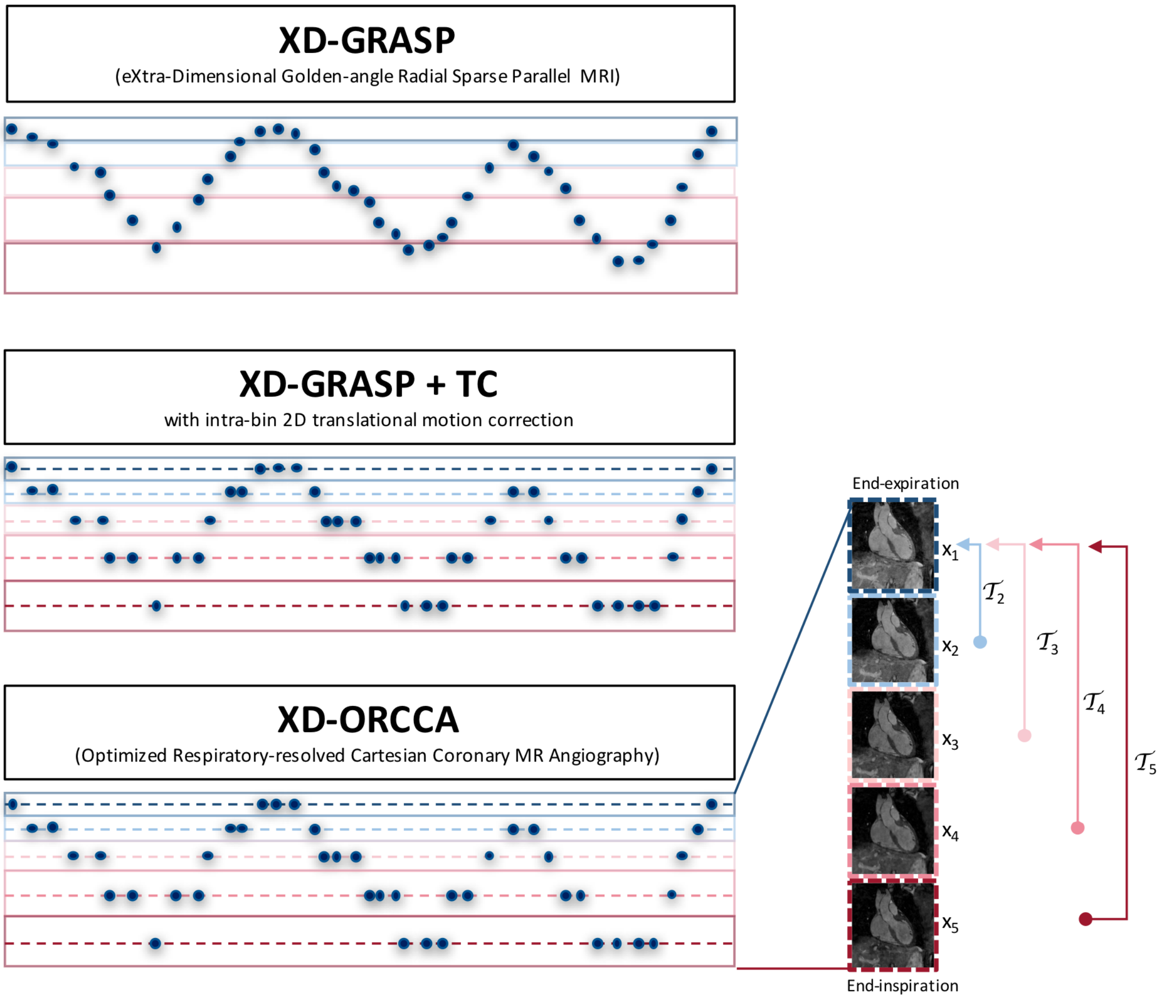

Supporting Figure S1 *Schematic representation of XD-GRASP (top), XD-GRASP with intrabin 2D translational motion correction (XD-GRASP+TC) (middle) and XD-ORCCA (bottom). The dots represent the respiratory signal extracted from the image navigators (iNAVs), which corresponds to the estimated superior-inferior (SI) translational motion. For all methods, this information is used to separate the 3D coronary MRA (CMRA) data into 5 different respiratory phases (or bins), to reconstruct respiratory-resolved images. In XD-GRASP, respiratory-resolved images are reconstructed by exploiting total variation sparsity in the respiratory dimension. In XD-GRASP + TC, the 3D CMRA data within each bin are corrected for 2D translational motion to the center of each bin, to reduce residual intrabin motion. In XD-ORCCA, 2D translational motion correction within each bin is performed in k-space before the reconstruction (as in XD-GRASP+TC). Furthermore, intrabin motion corrected images (x_b) are aligned (using the 2D translational transform $\mathcal{T}_b$) to 1 respiratory position (end-expiration) to further increase sparsity in the respiratory dimension.*

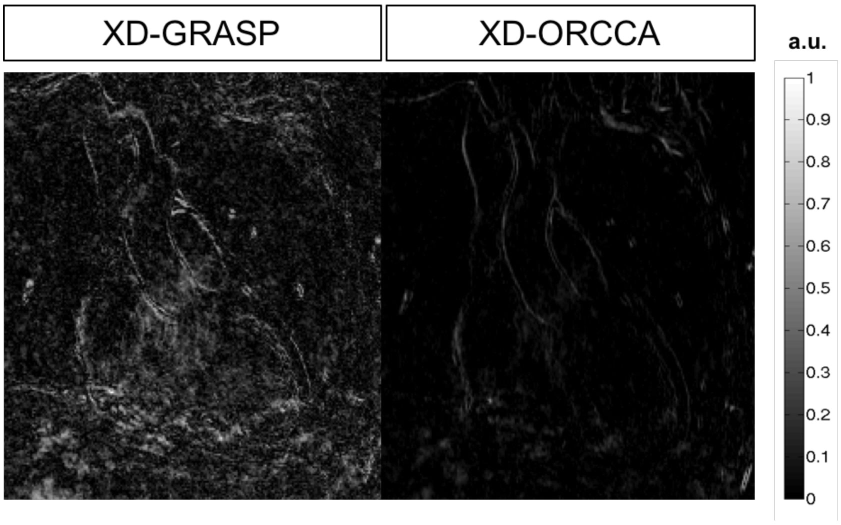

Supporting Figure S2 *Example of temporal sparsity achieved with XD-GRASP (left) and XD-ORCCA (right). The proposed XD-ORCCA increases the sparsity in the respiratory dimension by incorporating translational motion information into the sparsifying operator along the temporal dimension. Translational information is extracted from 2D interleaved iNAVs.*

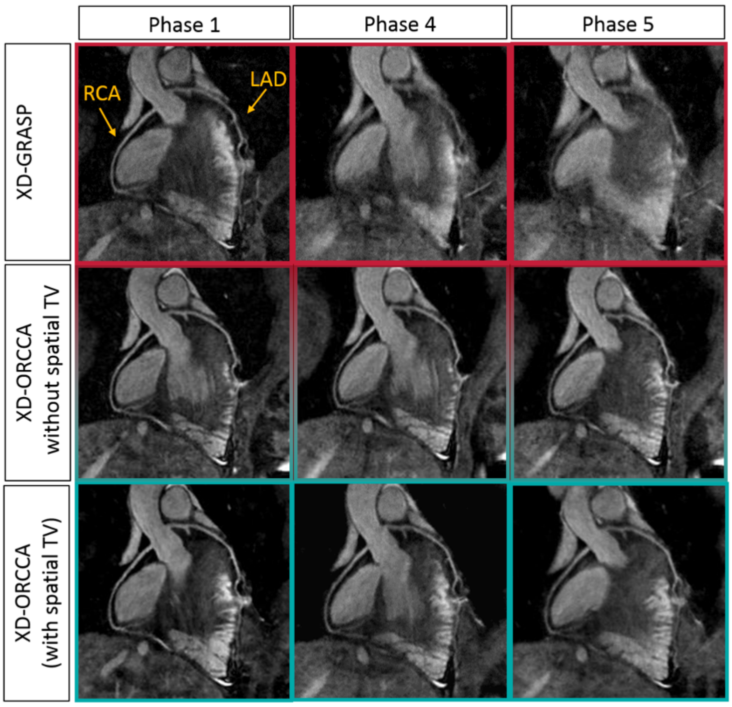

Supporting Figure S3 *Reformatted respiratory-resolved images obtained for 1 representative subject using XD-GRASP (top), XD-ORCCA without spatial TV regularization (middle), and XD-ORCCA with spatial TV regularization (bottom). For each method, respiratory phases 1 (end-expiration), 4 and 5 (end-inspiration) are shown. Each image shows the right coronary artery (RCA) and the left anterior descending coronary artery (LAD). Including spatial TV regularization in XD-ORCCA slightly improved the quality of the respiratory-resolved images, because of its denoising effect. Hence, the use of translational motion to further increase the sparsity in the respiratory dimension seems to explain a significant part of the improvement in image quality of the proposed XD-ORCCA in comparison to XD-GRASP.*

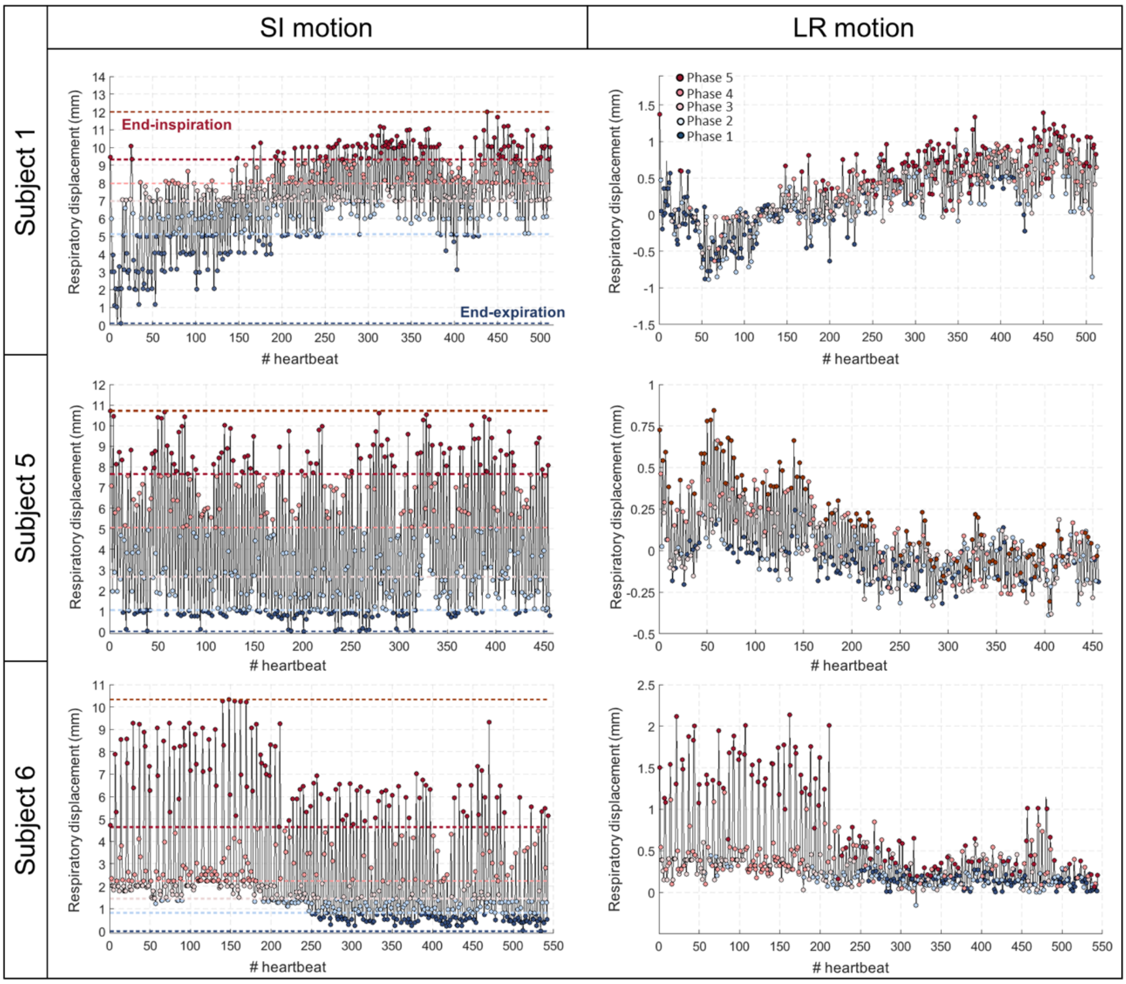

Supporting Figure S4 *Respiratory displacement values at each heartbeat, obtained from the 2D image navigators along the SI (left) and left-right (LR) (right) directions, for 3 representative subjects. The end-expiration SI position is used as reference. The horizontal lines (SI plots) and colors represent the different respiratory phases. Subject 5 had a regular breathing pattern with small LR displacements (maximum of 1.23 mm). For this subject, XD-GRASP provided a good end-expiration image, as the SI bin width was 1.05 mm and the maximum LR displacement was 0.56 mm. However, for phase 4 and 5 the SI bin width was approximately 3 mm, and hence, blurring motion is visible in the corresponding XD-GRASP images. Subject 6 presented a respiratory drift for the first 200 heartbeats. Nevertheless, the end-expiration images obtained with XD-GRASP have good quality, as the SI bin width was 0.81 mm and the maximum LR displacement for that bin was 0.29 mm. However, for the end-inspiration phase, the SI bin width was 5.69 mm and the LR displacement was 2.07 mm. This large residual intrabin motion deteriorates the quality of end-inspiration XD-GRASP images. Subject 1 showed a more irregular breathing pattern and a substantial respiratory drift, which resulted in a large end-expiration bin of 5.12 mm width. In addition, LR motion was present in all the respiratory phases (about 1.6 mm). For this case, XD-GRASP images were substantially degraded by respiratory motion. The XD-ORCCA approach provided high-quality respiratory-resolved images even for subject 1, which had a more irregular breathing pattern. For all 10 subjects, the overall SI respiratory bin widths for phases 1 to 5 were 1.55 $\pm$ 1.35 mm, 1.17 $\pm$ 0.54 mm, 1.61 $\pm$ 0.91 mm, 2.78 $\pm$ 1.04 mm and 5.88 $\pm$ 3.40 mm, respectively. The overall LR displacements for phases 1 to 5 were 1.01 $\pm$ 0.55 mm, 1.29 $\pm$ 0.56 mm, 1.16 $\pm$ 0.45 mm, 1.43 $\pm$ 0.50 mm and 1.80 $\pm$ 0.64 mm, respectively.*

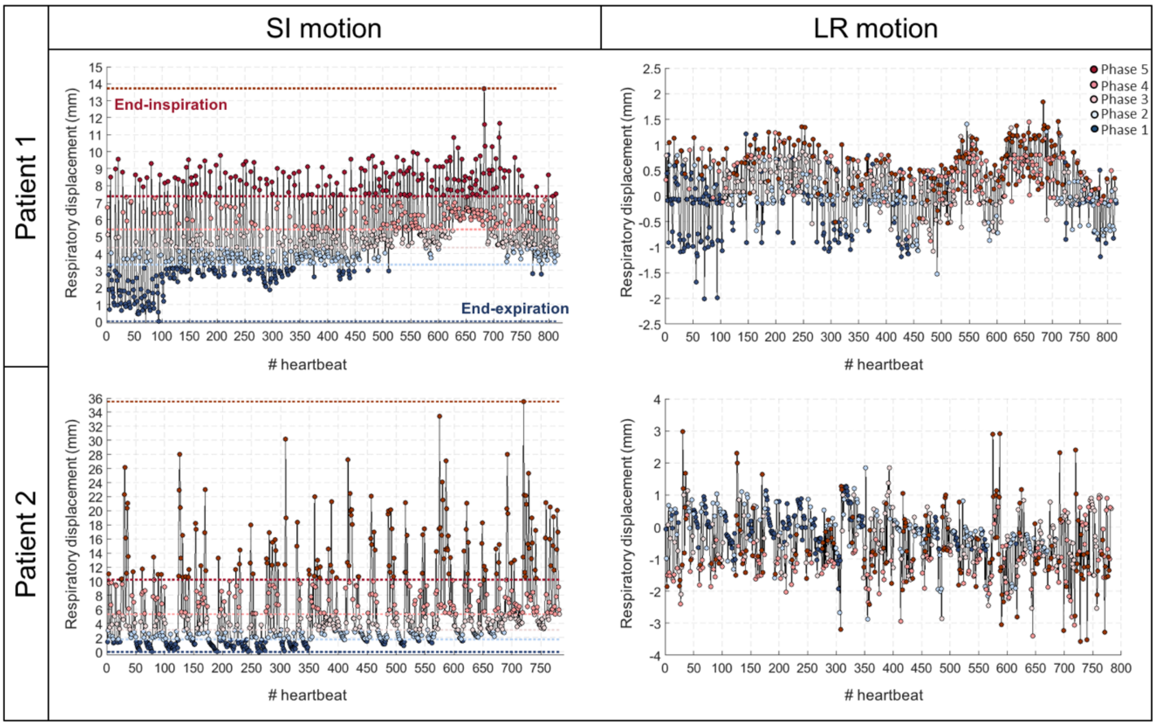

Supporting Figure S5 *Respiratory displacement values at each heartbeat, obtained from the 2D image navigators along the SI (left) and LR (right) directions, for 2 patients with cardiovascular disease. The end-expiration SI position is used as reference. The horizontal lines (SI plots) and colors represent the different respiratory phases. Patient 1 presented a large respiratory drift. Patient 2 had an irregular breathing pattern, a slight respiratory drift and large SI motion displacements. For patients 1 and 2, the respiratory phase with the smallest SI displacement (phase 2) only had a maximum displacement of 1.07 and 1.37 mm, respectively. However, the corresponding LR displacements were 2.92 and 4.72 mm. Cartesian XD-GRASP does not provide good-quality images in the presence of residual intrabin respiratory motion, particularly if this motion is in the LR direction. The XD-ORCCA method produces images that allow the visualization of the coronary arteries in both patients.*
